# Prediction of Non-Cardiac Organ Failure in Acute Myocardial Infarction Patients with Arrhythmia: A Retrospective Case–Control Study

**DOI:** 10.3390/jcm14217667

**Published:** 2025-10-29

**Authors:** Luqin Yan, Bowen Zhang, Boyu Chen, Yang Yan, Tao Shi

**Affiliations:** 1 Department of Cardiovascular Surgery, The First Affiliated Hospital of Xi’an Jiaotong University, No. 277 Yanta West Road, Xi’an 710061, China; yanluqin@xjtu.edu.cn (L.Y.);; 2 School of Public Health, Xi’an Jiaotong University, Xi’an 710061, China; 3 Health Science Center, Xi’an Jiaotong University, Xi’an 710061, China

**Keywords:** acute myocardial infarction, arrhythmia, organ failure, LASSO regression, SHAP, nomogram

## Abstract

**Background:** Non-cardiac organ failure is a severe complication following acute myocardial infarction (AMI), particularly among patients with concomitant arrhythmia. This study aimed to identify risk factors at admission that were associated with in-hospital non-cardiac organ failure. **Methods:** This case–control study enrolled AMI patients hospitalized for treatment with any type of arrhythmia. Patients were divided into the complication group and the control group based on the development of non-cardiac organ failure. Relaxed least absolute shrinkage and selection operator (LASSO) logistic regression and multivariate logistic regression were performed to identify risk factors, which were subsequently used to develop a predictive model. Shapley Additive Explanation (SHAP) values were applied to enhance model interpretability. **Results:** A total of 668 patients were enrolled, including 59 individuals in the complication group. After LASSO-logistic and multivariate logistic regression, five independent risk factors were identified and ranked by their SHAP values: Killip class III/IV [odds ratio (OR)] = 2.409, 95% confidence interval (CI): 1.246–4.657, *p* = 0.009], fibrin degradation products [OR = 1.029, 95% CI: 1.009–1.049, *p* = 0.003], N-terminal pro-B-type natriuretic peptide [OR = 1.000, 95% CI: 1.000–1.000, *p* = 0.002], type 2 diabetes mellitus [OR = 1.888, 95% CI: 1.005–3.546, *p* = 0.048], and cardiogenic shock [OR = 3.443, 95% CI: 1.463–8.089, *p* = 0.005]. The model demonstrated good discriminative ability with an area under the curve of 0.790 (95% CI: 0.720–0.861). Internal validation showed a calibration slope of 0.953 and a Brier score of 0.067, indicating strong overall predictive accuracy. **Conclusions:** This study identified five independent risk factors associated with in-hospital non-cardiac organ failure in AMI patients with arrhythmia. The nomogram might assist in early risk stratification, ultimately improving clinical outcomes in high-risk AMI patients with arrhythmia.

## 1. Introduction

Acute myocardial infarction (AMI) is a rapidly progressing, severe, and life-threatening manifestation of coronary artery disease, most commonly triggered by the rupture of coronary atherosclerotic plaques, leading to succeeding thrombus formation and vessel occlusion [1]. Arrhythmias are common complications of AMI, requiring prompt recognition and timely intervention [2,3]. The presence of arrhythmia, particularly ventricular fibrillation (VF), is strongly associated with increased mortality among AMI patients, with VF being the leading cause of early death [4,5]. One study indicated that although the incidence of in-hospital VF and mortality following AMI have significantly declined over the past two decades, the mortality risk among AMI patients who develop VF remains approximately 10-fold higher than that of patients without this arrhythmic complication [6]. Moreover, VF is also independently related to an increased risk of 30-day mortality [7]. Therefore, the management of AMI patients who develop arrhythmias during hospitalization remains a critical clinical challenge and warrants special attention.

Emerging evidence highlights that AMI is not confined to cardiac injury, but it is also associated with non-cardiac organ failure, especially prevalent in patients with arrhythmia. AMI can induce hemodynamic alterations, including left ventricular diastolic and systolic dysfunction, reduced cardiac output, and insufficient peripheral perfusion, which may result in acute kidney injury (AKI) or hepatic congestion. The thromboembolic events triggered by AMI are also linked to complications, such as ischemic hepatitis and stroke [8]. Furthermore, the ischemic injury in AMI initiates a cascade of pathological events, including oxidative stress, inflammatory responses, and apoptosis [9]. The subsequent release of pro-inflammatory cytokines (e.g., tumor necrosis factor-α, interleukin-6) and cardiac-specific biomarkers (e.g., troponins) can lead to microvascular dysfunction and neurohormonal activation, which may further contribute to remote multi-organ dysfunction [1].

Generally, determine the related risk factors for non-cardiac organ failure in patients with AMI complicated with arrhythmia, which may improve the prediction and prevention of organ injury in this high-risk population.

## 2. Materials and Methods

### 2.1. Participants Enrollment

**Figure 1:**
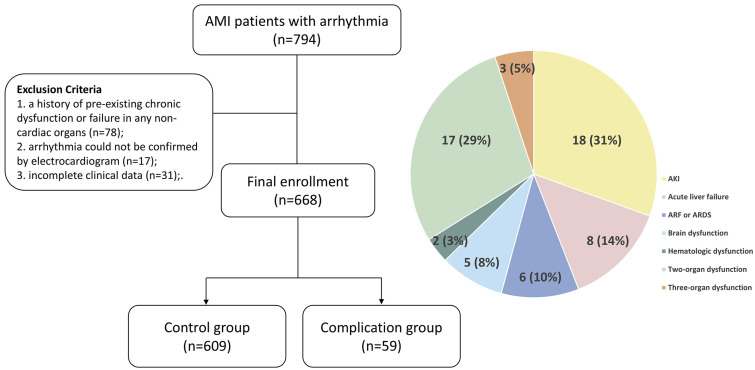
Flowchart of this study with the separation of the case group. AKI, acute kidney injury; ARF, acute respiratory failure; ARDS, acute respiratory distress syndrome.

This case–control investigation consecutively recruited acute myocardial infarction (AMI) patients admitted to the Cardiovascular Department of the First Affiliated Hospital of Xi’an Jiaotong University. Inclusion criteria comprised: (1) AMI diagnosis validated through established clinical guidelines; (2) electrocardiographically documented arrhythmias requiring therapeutic intervention upon admission, including sinus bradycardia, atrial flutter, atrial fibrillation, supraventricular tachycardia, premature ventricular complexes, ventricular tachycardia, ventricular fibrillation, and atrioventricular block. Exclusion parameters involved: (1) pre-existing chronic dysfunction in non-cardiac organ systems; (2) transient or self-resolving arrhythmic episodes lacking definitive electrocardiographic confirmation; (3) insufficient clinical documentation precluding statistical evaluation. The flowchart of this study is shown in Figure 1.

The research protocol received ethical authorization from the Institutional Review Board at Xi’an Jiaotong University’s First Affiliated Hospital (XJTU1AF2025LSYY-610, Date: 15 July 2025), adhering strictly to Helsinki Declaration principles. In light of the retrospective observational methodology employed, the ethics governance body formally exempted participant consent requirements for this non-interventional analytical study.

### 2.2. Data Collection and Grouping

Clinical data were obtained from the Biobank of the First Affiliated Hospital of Xi’an Jiaotong University. Baseline characteristics included demographic information (e.g., age and gender), medical history, biochemical laboratory tests (e.g., complete blood count, liver function tests, and renal function tests), imaging findings (echocardiography), and electrocardiogram results at admission. The short-term outcomes assessed included in-hospital mortality and length of stay, as well as the use of and extracorporeal life support devices.

According to whether non-cardiac organ failure occurred during hospitalization, the patients were divided into complication group and control group. The non-cardiac organ failure included the following entities: (1) AKI [10,11]; (2) acute liver failure [12]; (3) acute respiratory failure or acute respiratory distress syndrome [13]; (4) hematologic dysfunction, including thrombocytopenia and acquired coagulation factor deficiency; (5) transient organic psychotic conditions, anoxic brain injury or acute encephalopathy.

### 2.3. Statistical Analysis

Statistical preprocessing involved the exclusion of variables demonstrating missing data exceeding 20%. The retained variables underwent imputation through multiple imputation by chained equations with 5 cycles, with the fifth imputation dataset retained for further modeling. Normally distributed continuous parameters were expressed as mean ± standard deviation and analyzed through Student’s *t*-tests. Nonparametric continuous variables were reported as median (interquartile range) with Mann–Whitney U test implementation. Categorical measures were quantified as absolute counts (percentages), with intergroup comparisons executed via χ^2^ test or Fisher’s exact test based on expected frequencies.

Feature selection employed least absolute shrinkage and selection operator (LASSO) regression coupled with multivariate logistic regression to identify independent predictors demonstrating preliminary associations (*p* < 0.10). Shapley Additive Explanations (SHAP) values were calculated to quantify individual predictor contributions within the LASSO-derived model, subsequently integrated into a clinical prediction nomogram. Model discrimination was assessed through receiver operating characteristic analysis (AUC quantification), calibration curves, and decision curve analysis. Internal validation utilized bootstrap resampling with 1000 replicates, with model calibration evaluated through calibration slopes and Brier scores.

All computations were performed utilizing SPSS (version 27.0) and R (version 4.5.1). Statistical significance threshold was established at two-tailed *p* < 0.05.

## 3. Results

### 3.1. Baseline Characteristics

**Figure 2:**
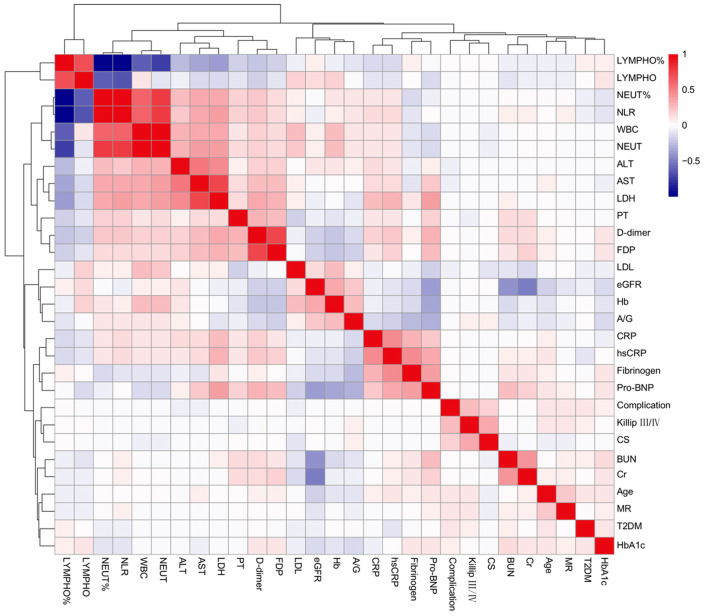
Heatmap of all variables with statistical differences in baseline. Abbreviations as in Table 1.

The final cohort comprised 668 eligible participants, with detailed stratification of the 59 complication group subjects illustrated in Figure 1. Baseline comparisons demonstrated significantly elevated rates of Killip class III/IV, type 2 diabetes mellitus (T2DM), pre-admission cardiogenic shock (CS), and mitral regurgitation in the complication cohort versus controls (all *p* < 0.05). Quantitative analysis revealed marked elevations in the complication group for age, leukocyte count, neutrophil count/percentage, neutrophil-to-lymphocyte ratio, high-sensitivity C-reactive protein, alanine aminotransferase, aspartate aminotransferase, creatinine, blood urea nitrogen, D-dimer, fibrin degradation products (FDP), lactate dehydrogenase, and pro-brain natriuretic peptide (pro-BNP) (all *p* < 0.05). Conversely, hemoglobin concentration, lymphocyte count/percentage, albumin-to-globulin ratio, and estimated glomerular filtration rate were significantly reduced in the complication group relative to controls (all *p* < 0.05, Table 1). Variables demonstrating associations below the *p* < 0.10 threshold were graphically presented in Figure 2.

Clinical outcome disparities included substantially higher utilization of intra-aortic balloon pump [11 (18.6%) vs. 35 (5.7%), *p* < 0.001] and extracorporeal membrane oxygenation [6 (10.2) vs. 7 (1.1), *p* < 0.001] in the complication group. Additionally, this cohort exhibited prolonged hospitalization duration [6 (4, 8) vs. 4 (3, 6), *p* < 0.001] compared to controls.

### 3.2. LASSO-Logistic and Multivariate Logistic Regression Results

**Figure 3:**
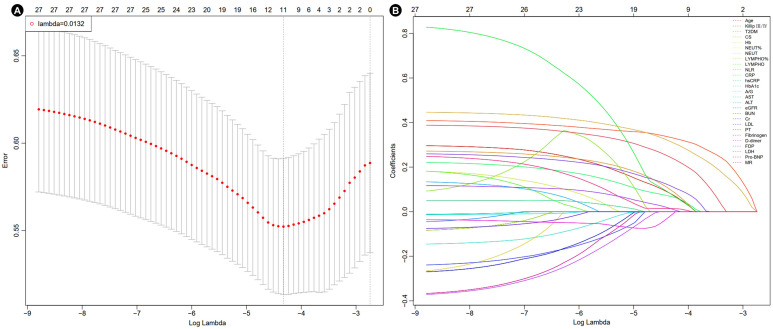
Cross-validation plot (**A**) and selection process plot (**B**) of LASSO-logistic regression. Abbreviations as in Table 1. The dashed line indicated the lambda.min = 0.0132, with 11 factors identified.

LASSO-logistic regression was primarily used to screen for risk factors. When lambda.min = 0.0132 and log (lambda.min) = −4.3275, 11 factors were identified (Figure 3 and Table 2). These 11 factors were further analyzed using multivariate logistic regression. Finally, five independent risk factors were settled, including Killip class III/IV [odds ratio (OR) = 2.409, 95% confidence interval (CI): 1.246–4.657, *p* = 0.009], T2DM [OR = 1.888, 95% CI: 1.005–3.546, *p* = 0.048], CS [OR = 3.443, 95% CI: 1.463–8.089, *p* = 0.005], FDP [OR = 1.029, 95% CI: 1.009–1.049, *p* = 0.003] and Pro-BNP [OR = 1.000, 95% CI: 1.000–1.000, *p* = 0.002] (Table 2).

### 3.3. SHAP Analysis and Construction of Nomogram

**Figure 4:**
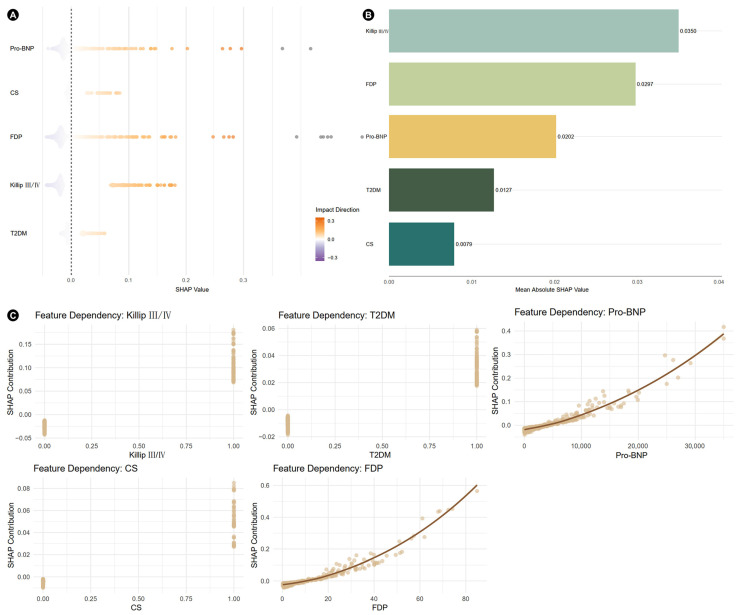
Beeswarm plot (**A**), bar plot (**B**) and feature dependency plots (**C**) of SHAP analysis. SHAP, Shapley additive explanations; other abbreviations as in Table 1.

The SHAP analysis was performed to interpret the contribution of each variable to the prediction of non-cardiac organ failure. Variables were ranked according to their mean absolute SHAP values, including Killip class III/IV (|SHAP| value = 0.0350), FDP (|SHAP| value = 0.0297), Pro-BNP (|SHAP| value = 0.0202), T2DM (|SHAP| value = 0.0127) and CS (|SHAP| value = 0.0079). SHAP values also illustrated both the direction and magnitude e of the contribution of each feature to the predicted risk (Figure 4).

**Figure 5:**
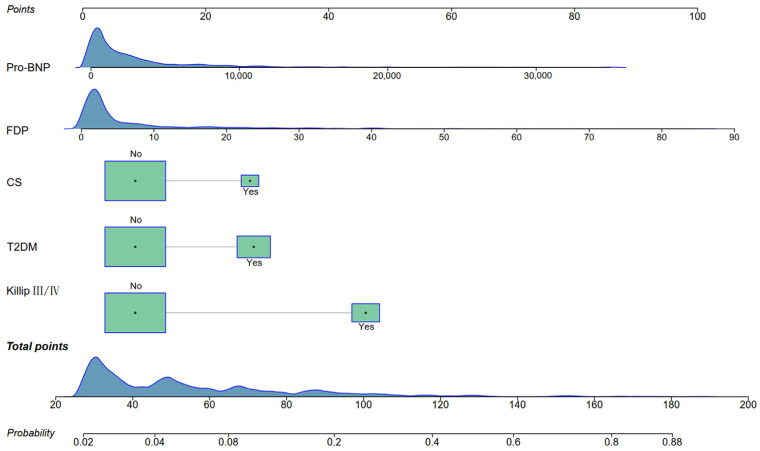
Nomogram to predict the risk of non-cardiac organ failure in AMI patients with arrhythmia. Abbreviations as in Table 1.

**Figure 6:**
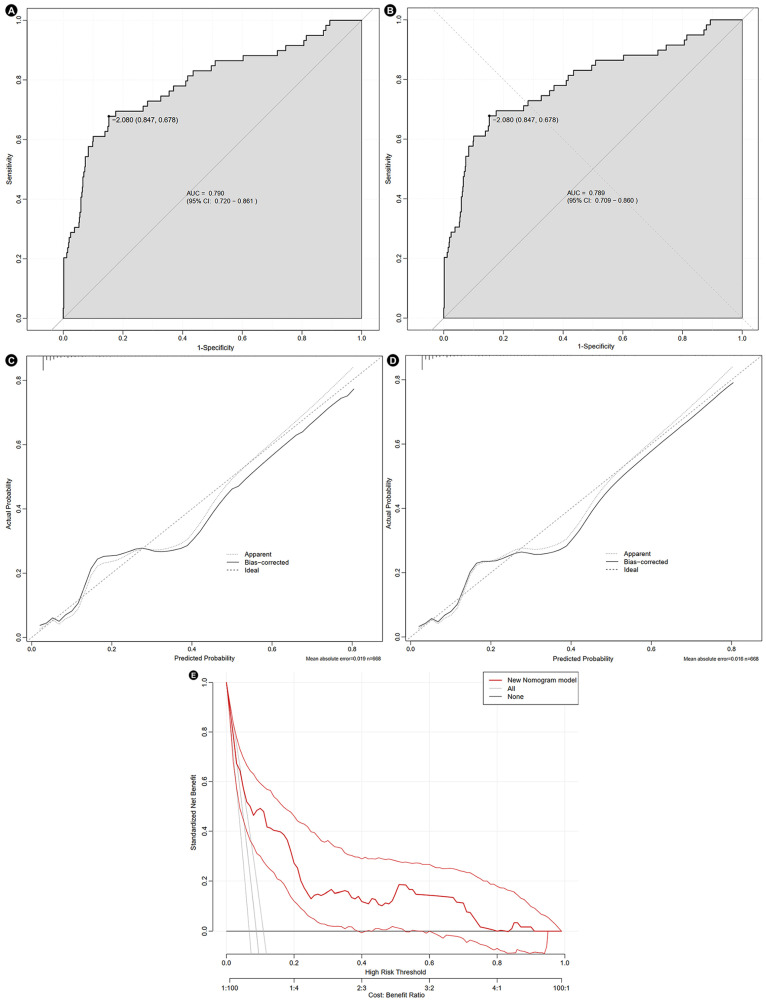
Evaluation of the nomogram. Receiver operating characteristic curve before (**A**) and after internal validation (**B**), calibration curve before (**C**) and after internal validation (**D**), and decision curve (**E**). AUC, area under the curve; CI, confidence intervals.

The nomogram based on these five factors is presented in Figure 5. The model demonstrated good discriminative performance, with an original AUC of 0.790 (95% CI: 0.720–0.861). The decision curve analysis revealed a positive net benefit across a range of high-risk probability thresholds. The calibration plot showed that the predicted probability is in good agreement with the observed probability. Following internal validation, the AUC remained robust at 0.789 (95% CI: 0.709–0.860). The calibration slope was 0.953, indicating good calibration; the Brier Score was 0.067, further supporting the good overall accuracy (Figure 6).

## 4. Discussion

This study aimed to evaluate whether the occurrence of arrhythmias was associated with a broader systemic vulnerability or generalized organ damage, which might reflect severe hemodynamic instability, prolonged hypoxia, or systemic inflammatory activation. This study enrolled 668 AMI patients with arrhythmia and stratified them into complication and control groups based on the presence of non-cardiac organ failure. Using LASSO-logistic and multivariate logistic regression analyses, Killip class III/IV, T2DM, CS, FDP, and pro-BNP were identified as independent risk factors for non-cardiac organ failure. SHAP analysis further elucidated the relative contribution of each variable to the predictive model. A nomogram incorporating these five parameters demonstrated good predictive accuracy and clinical utility. These findings offered a reliable framework for predicting non-cardiac organ failure in AMI patients with arrhythmia, supporting early clinical intervention and improved management strategies.

The pathophysiological cascade of AMI involves a progressive decline in cardiac output, ultimately leading to systemic hypoperfusion and non-cardiac organ failure, such as kidneys, liver, and brain [14,15,16]. Cardiogenic shock represents the most severe hemodynamic complication of AMI. AKI, a well-documented comorbidity, arises from multiple mechanisms, including renal hypoperfusion, systemic inflammatory responses, right ventricular failure, and complications related to mechanical circulatory support. Respiratory complications such as ARF and ARDS, as well as hepatic dysfunction including acute liver injury, frequently arise as a result of hemodynamic instability and the associated metabolic disturbances. Meanwhile, in the context of AMI, cardiac arrest may occur, leading to initial cerebral ischemia followed by reperfusion after successful resuscitation. This sequence of events can result in post-cardiac arrest brain injury, which is also the leading cause of disability and mortality among patients who survive the immediate aftermath of cardiac arrest [17].

Clinical evidence highlights the critical impact of non-cardiac organ dysfunction in AMI outcomes. Patients experiencing AKI during acute coronary syndrome retain elevated risks of recurrent AKI and progression to chronic kidney disease, even after apparent renal function recovery [16]. Those with prior AKI episodes demonstrate increased in-hospital mortality and higher rates of major cardiovascular complications both during hospitalization and over a 5-year follow-up period [18]. Concurrently, AMI patients with acute liver injury exhibit greater susceptibility to coagulation disorders than those with normal liver function, while also facing independent mortality risks at multiple timepoints (in-hospital, 28-day, and 90-day) [19].

This study revealed five independent risk factors for in-hospital non-cardiac organ failure for AMI patients with arrhythmia. Previous studies have demonstrated that Killip Class IV and T2DM independently heighten the risk of developing renal dysfunction in AMI patients during a 6-month follow-up [20]. T2DM can induce microvascular damage through progressive endothelial dysfunction, which is initially reversible but may ultimately lead to irreversible organ injury, including nephropathy, liver dysfunction, neuropathy, and cognitive impairment [21,22]. These chronic damages may also increase the susceptibility of multiple organs to acute injury.

Killip class III/IV and CS both reflect significant impairment of cardiac function in AMI patients and are more frequently observed in patients presenting with arrhythmia. Although left ventricular ejection fraction was not identified as an independent risk factor in this study, arrhythmias are more common in patients who do not receive timely reperfusion, particularly those who have already developed reduced left ventricular ejection fraction [2]. This observation is consistent with the presence of advanced hemodynamic compromise, as indicated by Killip class III/IV or CS. Furthermore, the coexistence of arrhythmia may further deteriorate cardiac function. One study reported a higher prevalence of Killip class III/IV among patients with new-onset right bundle branch block, which may in turn contribute to increased in-hospital mortality [23]. In addition, T2DM may further increase the risk of in-hospital mortality and cerebrovascular complications in AMI patients complicated by CS [24].

While the precise role of FDP in organ injury remains incompletely elucidated, their association with coagulopathy and thromboembolic events suggests a multifaceted involvement in systemic organ dysfunction. Previous studies have shown that heightened D-dimer levels are linked to an increased risk of AKI in male patients, but not in female patients, further highlighting the potential gender-specific relationship between coagulation-related biomarkers and non-cardiac organ failure [25]. Pro-BNP, a stable biomarker of ventricular strain, is closely correlated with the extent of myocardial infarction and reflects hemodynamic instability [26]. One study has indicated that elevated Pro-BNP levels may predict the incidence of postoperative AKI in patients with pre-existing renal impairment undergoing cardiac surgery [27]. The impact of Pro-BNP on dysfunction of other organ systems also deserves further investigation.

This study also has several limitations. First, the investigation was limited by its single-center, retrospective design, modest sample size, and incomplete data collection. Second, due to the limited sample size of the complication group, it was unable to further differentiate between types of arrhythmias. Different arrhythmia subtypes may exert distinct effects on non-cardiac organ failure, and the underlying pathways for individual organ failures may differ—both aspects warrant further investigation in future studies. Future multicenter studies with larger cohorts are warranted to better elucidate the impact of AMI on non-cardiac organ dysfunction and to support more comprehensive patient management strategies.

## 5. Conclusions

This study identified five independent risk factors associated with in-hospital non-cardiac organ failure in AMI patients with arrhythmia: Killip class III/IV, FDP, Pro-BNP, T2DM, and CS. These factors may reflect global physiological derangement and contribute to non-cardiac organ susceptibility. Based on these variables, a predictive nomogram was developed, demonstrating favorable discrimination and calibration performance. The incorporation of SHAP values further improved the interpretability of the model by illustrating the relative impact of each variable on the outcome. This risk prediction tool holds significant potential for clinical application, enabling early recognition of high-risk patients, timely intervention, and the realization of more personalized management strategies.

## Figures and Tables

**Table 1:** Baseline characteristics and in-hospital outcomes of complication and control groups.

Items	Total (*n* = 668)	Complication Group (*n* = 59)	Control Group (*n* = 609)	*p* Value
Age (years)	67 (57, 74)	70 (60, 76)	66 (57, 73)	0.018
Gender (male, %)	514 (76.9)	42 (71.2)	472 (77.5)	0.271
BMI (kg/m^2^)	24.45 (22.29, 26.01)	24.42 (21.13, 26.43)	24.45 (22.49, 25.97)	0.510
Smoking (%)	201 (30.1)	22 (37.3)	179 (29.4)	0.207
Drinking (%)	71 (10.6)	4 (6.8)	67 (11.0)	0.315
STEMI (%)	391 (5835)	36 (61.0)	355 (58.3)	0.685
Killip III/IV (%)	115 (17.2)	28 (47.5)	87 (14.3)	<0.001
**Comorbidities (%)**				
Hypertension	511 (76.5)	49 (83.1)	462 (75.9)	0.214
T2DM	154 (23.1)	22 (37.3)	132 (21.7)	0.007
CS	52 (7.8)	13 (22.0)	39 (6.4)	<0.001
Previous AF	37 (5.5)	4 (6.8)	33 (5.4)	0.890
**Arrhythmia (%)**				
Sinus bradycardia	34 (5.1)	1 (1.7)	33 (5.4)	0.350
Atrial flutter	23 (3.4)	3 (5.1)	20 (3.3)	0.447
AF	300 (44.9)	31 (52.5)	269 (44.2)	0.217
Supraventricular tachycardia	35 (5.2)	2 (3.4)	33 (5.4)	0.760
PVC	165 (14.7)	10 (16.9)	155 (25.5)	0.148
Ventricular tachycardia	116 (17.4)	13 (22.0)	103 (16.9)	0.321
VF	77 (11.5)	7 (11.9)	70 (11.5)	0.932
Atrioventricular block	107 (16.0)	11 (18.6)	96 (15.8)	0.565
**Biochemical results**				
Hb (g/L)	139.00 (127.00, 150.00)	132.00 (116.00, 143.00)	140.00 (128.00, 150.00)	0.003
WBC (10^9^/L)	9.06 (6.87, 12.11)	9.95 (7.34, 15.02)	8.92 (6.83, 11.99)	0.044
NEUT (10^9^/L)	6.96 (4.73, 9.94)	8.54 (5.49, 12.55)	6.90 (4.66, 9.83)	0.015
NEUT% (%)	77.00 (69.50, 85.48)	83.50 (71.70, 89.10)	76.70(69.30, 85.10)	0.006
LYMPHO (10^9^/L)	1.32 (0.96, 1.80)	1.10 (0.76, 1.60)	1.33 (0.99, 1.82)	0.008
LYMPHO% (%)	15.69 (9.42, 22.16)	11.21 (6.29, 20.39)	15.88 (9.72, 22.23)	0.003
NLR	4.93(3.18, 9.09)	7.45 (3.41, 13.97)	4.78 (3.17, 8.77)	0.003
CRP (mg/L)	16.10 (10.00, 45.73)	21.27 (10.00, 73.40)	15.83 (10.00, 44.07)	0.077
hs-CRP (mg/L)	4.48 (1.55, 9.55)	7.64 (2.24, 10.00)	4.31 (1.52, 9.43)	0.020
HbA1c (%)	5.94 (5.60, 6.80)	6.20 (5.70, 7.79)	5.90 (5.60, 6.66)	0.054
ALT (U/L)	33.00 (22.00, 56.00)	52.00 (22.00, 119.00)	33.00 (22.00, 53.46)	0.003
AST (U/L)	47.85 (27.00, 114.00)	70.00 (27.00, 201.00)	47.00 (27.00, 109.29)	0.077
Total protein (g/L)	62.65 (58.55, 67.10)	62.30 (58.30, 67.40)	62.70 (58.60, 67.10)	0.824
Albumin (g/L)	37.30 ± 4.82	36.29 ± 4.48	37.40 ± 4.85	0.104
Globulin (g/L)	25.50 (23.20, 28.10)	26.50 (23.50, 29.40)	25.40 (23.10, 28.00)	0.183
A/G	1.44 (1.28, 1.65)	1.37 (1.17, 1.48)	1.44 (1.29, 1.66)	0.007
eGFR (mL/min/1.73 m^2^)	87.80 (71.57, 99.36)	59.65 (38.81, 79.18)	89.00 (74.68, 99.97)	<0.001
Cr (umol/L)	69.00 (57.00, 84.00)	98.00 (78.00, 130.00)	68.00 (56.00, 80.50)	<0.001
BUN (mmol/L)	6.06 (4.85, 7.48)	9.04 (6.09, 14.36)	6.00 (4.77, 7.23)	<0.001
LDL (mmol/L)	2.15 (1.60, 2.72)	1.91 (1.43, 2.63)	2.17 (1.64, 2.73)	0.063
HDL (mmol/L)	0.94 (0.81, 1.10)	0.92 (0.80, 1.10)	0.95 (0.81, 1.10)	0.889
TG (mmol/L)	1.07 (0.77, 1.54)	0.97 (0.63, 1.72)	1.08 (0.79, 1.54)	0.235
TC (mmol/L)	3.81 (3.13, 4.53)	3.59 (2.98, 4.38)	3.82 (3.16, 4.55)	0.206
APTT (s)	31.70 (27.20, 37.90)	31.90 (26.65, 38.30)	31.70 (27.20, 37.88)	0.943
PT (s)	13.45 (12.40, 14.40)	14.10 (11.70, 15.90)	13.40 (12.40, 14.40)	0.073
Fibrinogen (g/L)	3.23 (2.57, 4.13)	3.38 (2.78, 4.73)	3.2 (2.57, 4.07)	0.080
D-dimer (mg/L)	0.68 (0.40, 1.67)	1.27 (0.50, 3.63)	0.64 (0.40, 1.54)	0.001
FDP (mg/L)	2.48 (1.40, 6.89)	5.40 (1.73, 18.12)	2.40 (1.37, 6.29)	0.001
LDH (U/L)	298.00 (229.25, 468.00)	352.00 (262.00, 831.00)	296.00 (225.00, 454.50)	0.012
CK-MB (U/L)	28.00 (15.00, 81.00)	27.00 (15.00, 106.10)	28.00 (15.00, 80.75)	0.706
CK (U/L)	234.00 (99.00, 786.00)	254.00 (105.00, 939.00)	232.00 (97.75, 776.25)	0.652
hs-cTnT (ng/mL)	0.56 (0.10, 2.32)	0.84 (0.13, 2.99)	0.54 (0.09, 2.31)	0.200
Pro-BNP (pg/mL)	1302.50 (373.50, 3476.00)	3451.02 (1382.00, 10,783.00)	1170.00 (340.20, 3159.00)	<0.001
**Echo cardiology**				
LVEF (%)	48.00 (42.00, 59.00)	45.00 (43.00, 51.75)	48.50 (42.00, 59.00)	0.396
MR (%)	144 (21.6)	22 (37.3)	122 (20.0)	0.002
PAH (%)	63 (9.4)	7 (11.9)	56 (9.2)	0.503
**In-hospital outcomes**				
PCI (%)	555 (83.1)	46 (78.0)	509 (83.6)	0.272
IABP (%)	46 (6.9)	11 (18.6)	35 (5.7)	0.001
ECMO (%)	13 (1.9)	6 (10.2)	7 (1.1)	<0.001
Length of hospital stay (days)	4 (3, 7)	6 (4, 8)	4 (3, 6)	<0.001
In-hospital mortality (%)	16 (2.4)	4 (6.8)	12 (2.0)	0.063

BMI, body mass index; STEMI, ST-elevated myocardial infarction; T2DM, type 2 diabetes mellitus; AF, atrial fibrillation; VF, ventricular fibrillation; PVC, premature ventricular contraction; CS, cardiogenic shock; Hb, hemoglobin; WBC, white blood cell; NEUT, neutrophil count; LYMPHO, lymphocyte count; NLR, neutrophil-to-lymphocyte ratio; CRP, C-reactive protein; hs-CRP, hypersensitive C-reactive protein; HbA1c, glycosylated hemoglobin A1C; ALT: alanine aminotransferase; AST, aspartate aminotransferase; A/G, albumin-globulin ratio; eGFR, estimated glomerular filtration rate; Cr, creatinine; BUN, blood urea nitrogen; LDL, low-density lipoprotein; HDL, high-density lipoprotein; TG, triglycerides; TC, total cholesterol; APTT, activated partial thromboplastin time; PT, prothrombin time; FDP, fibrin degradation product; LDH, lactate dehydrogenase; CK-MB, creatine kinase isoenzymes MB; CK, creatine kinase; hs-cTnT, high-sensitive cardiac troponin; ProBNP, pro-brain natriuretic peptide; LVEF, left ventricular ejection fraction; MR, mitral regurgitation; PAH, pulmonary arterial hypertension; PCI, percutaneous coronary intervention; IABP, intra-aortic balloon pump; ECMO, extracorporeal membrane oxygenation.

**Table 2:** Results of LASSO-logistic regression and multivariate regression model.

Items	LASSO-Logistic Regression		Multivariate Logistic Regression	
	Assignment	Coefficient	OR (95% CI)	*p* Value
Age (years)	Male = 1, Famale = 0	0.2365	1.014 (0.985, 1.043)	0.359
Killip III/IV (%)	Yes = 1, No = 0	0.3987	2.409 (1.246, 4.657)	0.009
T2DM (%)	Yes = 1, No = 0	0.2703	1.888 (1.005, 3.546)	0.048
CS (%)	Yes = 1, No = 0	0.4426	3.443 (1.463, 8.089)	0.005
Hb (g/L)	Continuous variable			
WBC (10^9^/L)	Continuous variable			
NEUT (10^9^/L)	Continuous variable			
NEUT% (%)	Continuous variable			
LYMPHO (10^9^/L)	Continuous variable			
LYMPHO% (%)	Continuous variable			
NLR	Continuous variable	0.2637	1.027 (0.988, 1.068)	0.177
CRP (mg/L)	Continuous variable	0.1335	1.002 (0.995, 1.010)	0.541
hs-CRP (mg/L)	Continuous variable			
HbA1c (%)	Continuous variable			
A/G	Continuous variable			
ALT (U/L)	Continuous variable			
AST (U/L)	Continuous variable			
eGFR (mL/min/1.73 m^2^)	Continuous variable			
Cr (umol/L)	Continuous variable			
BUN (mmol/L)	Continuous variable			
LDL (mmol/L)	Continuous variable			
PT (s)	Continuous variable	0.2134	0.967 (0.867, 1.078)	0.545
Fibrinogen (g/L)	Continuous variable	0.0859	1.081 (0.862, 1.354)	0.501
D-dimer (mg/L)	Continuous variable			
FDP (mg/L)	Continuous variable	−0.2991	1.029 (1.009, 1.049)	0.003
LDH (U/L)	Continuous variable			
Pro-BNP (pg/mL)	Continuous variable	0.0180	1.000 (1.000, 1.000)	0.002
MR (%)	Yes = 1, No = 0	0.3813	1.809 (0.925, 3.537)	0.083

LASSO, least absolute shrinkage and selection operator; OR, odds ratio; CI, confidence interval; other abbreviations as in Table 1.

## Data Availability

Due to the privacy of the individuals involved in the research, the data in this article cannot be publicly shared. Upon reasonable request, the data will be shared with the corresponding author.

## Abbreviations

AMI	Acute myocardial infarction
AKI	Acute kidney injury
AUC	Area under the curve
CI	Confidence interval
CS	Cardiogenic shock
FDP	Fibrin degradation products
LASSO	Least absolute shrinkage and selection operator
OR	Odds ratio
Pro-BNP	Pro-brain natriuretic peptide
SHAP	Shapley additive explanations
T2DM	Type 2 diabetes mellitus
VF	Ventricular fibrillation
